# Benign Metastasizing Leiomyoma With Myomatous Erythrocytosis Syndrome: First Reported Coexistence as a Unified Hormonal Syndrome

**DOI:** 10.1002/rcr2.70687

**Published:** 2026-07-15

**Authors:** Venkatkiran Kanchustambham, Ashley McWalter, Oluwatobi E. Odetola, Recep Nigdelioglu

**Affiliations:** ^1^ Division of Pulmonary and Critical Care Medicine Sanford Health Fargo North Dakota USA; ^2^ Department of Internal Medicine University of North Dakota School of Medicine and Health Sciences Grand Forks North Dakota USA; ^3^ Division of Hematology Sanford Health Fargo North Dakota USA; ^4^ Department of Pathology Sanford Health Fargo North Dakota USA

**Keywords:** benign metastasizing leiomyoma, erythropoietin, HLRCC, hormonal syndrome, myomatous erythrocytosis syndrome, pulmonary nodules, secondary polycythemia, uterine leiomyoma

## Abstract

Benign metastasizing leiomyoma (BML) is a rare condition characterized by histologically benign smooth muscle tumours arising at extrauterine sites, most commonly the lungs, in women with a history of uterine leiomyomata. Erythropoietin (EPO)‐secreting uterine leiomyomata causing secondary erythrocytosis—termed myomatous erythrocytosis syndrome (MES)—is an exceedingly rare and underrecognized phenomenon first described by Thomson and Marson in 1953, with fewer than 70 cases reported to date. We report a 39‐year‐old woman who presented with incidentally discovered bilateral pulmonary nodules on imaging performed during workup for MES with markedly elevated serum EPO (26.7 mIU/mL). She had a longstanding history of uterine leiomyomata and endometriosis. CT‐guided biopsy of the dominant left lower lobe nodule (14 × 11 mm) confirmed BML: lesional cells were positive for oestrogen receptor (ER), progesterone receptor (PR), smooth muscle actin (SMA) and desmin, with retained fumarate hydratase (FH), excluding hereditary leiomyomatosis and renal cell carcinoma (HLRCC) syndrome. Total hysterectomy with bilateral salpingo‐oophorectomy (BSO) was performed; pathology confirmed multiple cellular leiomyomata without features of STUMP or leiomyosarcoma on University of Michigan expert consultation. Within 3 months of surgery, haemoglobin normalized to 14.2 g/dL and EPO to 6.7 mIU/mL, confirming the uterus as the source of ectopic EPO production. This case illustrates a novel unified hormonal syndrome in which uterine leiomyomata simultaneously drove pulmonary BML through ER/PR signalling and caused secondary polycythemia through ectopic EPO secretion. Hysterectomy/BSO resolved both processes concurrently, and this case highlights BML and MES as diagnoses to consider in premenopausal women with bilateral pulmonary nodules and unexplained erythrocytosis.

## Graphical abstract

Time course of haemoglobin (red, left axis) and serum erythropoietin (blue, right axis) before and after hysterectomy with bilateral salpingo‐oophorectomy (BSO, green vertical line, 31 July 2025), showing normalization of both parameters following tumour resection.

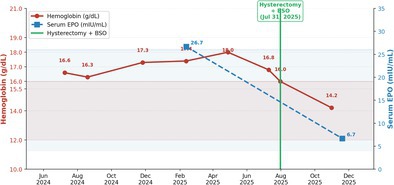

## 1 Introduction

Benign metastasizing leiomyoma (BML) is a rare entity first described by Steiner in 1939, characterized by the extrauterine spread of histologically benign smooth muscle tumours in women with a prior or concurrent history of uterine leiomyomata [1]. The lungs are the most common site of involvement, manifesting as single or multiple well‐circumscribed pulmonary nodules. Despite the benign histological appearance, pathogenesis remains debated, with prevailing theories supporting hematogenous or lymphatic dissemination of clonal uterine smooth muscle cells [2, 3].

The behaviour of BML is driven by oestrogen and progesterone receptor (ER/PR) expression, explaining its predilection for premenopausal women and documented regression following surgical menopause, GnRH agonist therapy or aromatase inhibitor use [4]. Malignant transformation is exceedingly rare, though exclusion of smooth muscle tumour of uncertain malignant potential (STUMP) and leiomyosarcoma requires expert pathological review.

Ectopic erythropoietin (EPO) secretion by uterine leiomyomata is an exceptionally rare and mechanistically distinct complication. Fewer than 20 cases have been reported in the world literature, and the coexistence of MES with pulmonary BML has, to our knowledge, not been previously described [5, 6].

We present a case in which both phenomena coexisted as a unified hormonal syndrome, with surgical menopause resolving the MES and providing a substrate for expected regression of pulmonary nodules.

## 2 Case Report

A 39‐year‐old woman of Korean ancestry, non‐smoker, presented to the pulmonary clinic for evaluation of incidentally discovered bilateral pulmonary nodules. She had no respiratory symptoms—specifically no cough, dyspnea, hemoptysis or chest pain. She denied fevers, night sweats or unintentional weight loss. There was no history of prior malignancy, occupational dust exposure, recent travel or exposure to endemic mycoses. She was adopted with no available biological family history.

Her medical history was significant for endometriosis and longstanding uterine leiomyomata. Polycythemia had been identified incidentally on routine bloodwork approximately 1 year prior. Serial complete blood counts demonstrated persistent erythrocytosis: haemoglobin peaked at 18.0 g/dL and haematocrit at 53.4%, with RBC counts consistently above 5.76 × 10^12^/L from July 2024 through April 2025 (Figure 4). Serum EPO was markedly elevated at 26.7 mIU/mL (February 2025), establishing MES—secondary (EPO‐driven) erythrocytosis—rather than primary polycythemia vera. JAK2 V617F mutation testing was negative. ANA, ANCA, anti‐MPO and anti‐PR3 antibodies were all negative.

CT of the abdomen and pelvis performed as part of polycythemia workup incidentally identified multiple bilateral pulmonary nodules and a markedly enlarged, bulky myomatous uterus with a dominant subserosal/broad ligament fibroid measuring 90 mm (Figure 1B). A dedicated CT of the chest demonstrated multiple bilateral noncalcified pulmonary nodules: the dominant lesion measured 14 × 11 mm in the left lower lobe, with remaining nodules approximately 5 mm, all well‐circumscribed and without lymphadenopathy, effusion or cavitation (Figure 1A).

**FIGURE 1:**
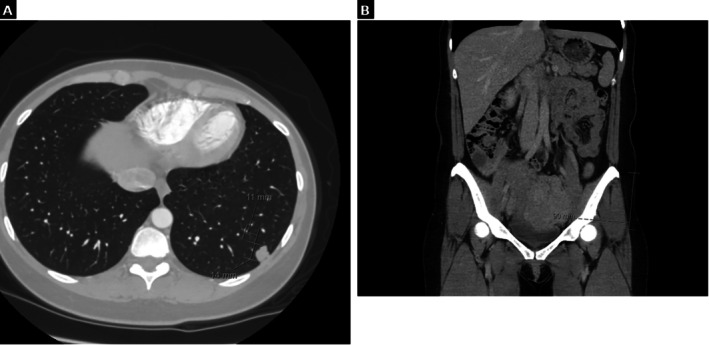
Computed tomography imaging. (A) Axial CT chest (lung window) demonstrating the dominant left lower lobe noncalcified pulmonary nodule measuring 14 × 11 mm (dashed measurement lines) with bilateral subcentimeter nodules consistent with benign metastasizing leiomyoma. (B) Coronal CT abdomen/pelvis (soft tissue window) demonstrating the markedly enlarged myomatous uterus with the dominant subserosal fibroid measuring 90 mm, representing the uterine primary source.

Given the clinical picture of bilateral pulmonary nodules in a premenopausal woman with known uterine leiomyomata, CT‐guided biopsy of the dominant left lower lobe nodule was performed. Histopathological examination revealed a spindle cell proliferation in intersecting fascicles without atypia, mitosis or necrosis (Figure 2A,B). Immunohistochemical (IHC) staining demonstrated diffuse strong positivity for desmin (Figure 2C), ER (Figure 2D) and PR (Figure 2E), with additional positivity for SMA and beta‐catenin (membranous). Markers for malignant spindle cell tumours were negative. Fumarate hydratase (FH) demonstrated retained expression, excluding HLRCC syndrome. The pathological findings were prepared and interpreted by R.N. The findings were consistent with BML of the lung.

**FIGURE 2:**
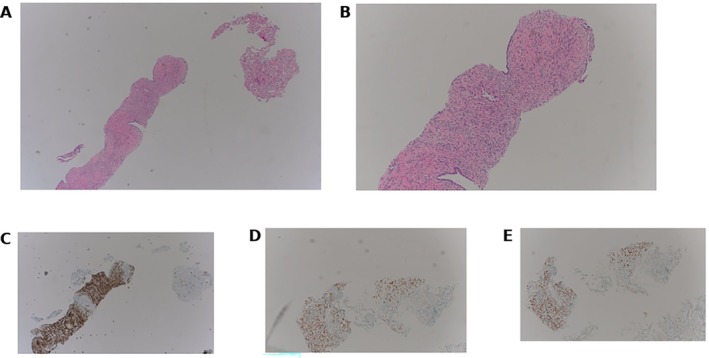
Histopathology of the left lower lobe CT‐guided biopsy confirming pulmonary benign metastasizing leiomyoma. (A) H&E low power: Core tissue involved by spindle cell neoplasm. (B) H&E high power: Bland spindle cells in intersecting fascicles without atypia, mitosis or necrosis. (C) Desmin immunohistochemistry (IHC): Diffuse strong cytoplasmic positivity confirming smooth muscle lineage. (D) Oestrogen receptor (ER) IHC: Nuclear positivity throughout lesional cells. (E) Progesterone receptor (PR) IHC: Nuclear positivity confirming hormone receptor‐driven BML.

Total hysterectomy with bilateral salpingo‐oophorectomy (BSO) was performed (July 31, 2025). The specimen weighed 563 g and contained more than 20 well‐circumscribed intramural nodules (0.4–2.5 cm). Histopathological examination demonstrated multiple cellular leiomyomata and a leiomyoma with bizarre nuclei, with low mitotic activity (2/10 HPF) and no necrosis (Figure 3A,B). IHC confirmed positivity for SMA and desmin (Figure 3C), negative 2SC (Figure 3D) and retained FH (Figure 3E), excluding HLRCC syndrome. Expert consultation by Drs. Rouba Ali‐Fehmi and Douglas Rottman at the University of Michigan confirmed no features of STUMP or leiomyosarcoma.

**FIGURE 3:**
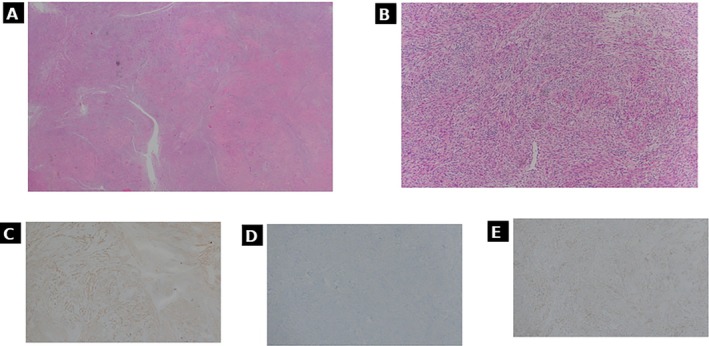
Histopathology of the hysterectomy specimen confirming the uterine primary. (A) H&E low power: Uterus with multiple spindle cell nodules (cellular leiomyomata). (B) H&E high power: Bland spindle cells morphologically identical to the pulmonary nodule, without atypia, mitosis or necrosis. (C) Desmin IHC: Positive in uterine leiomyomata. (D) 2‐succinocysteine (2SC) IHC: Negative, excluding fumarate hydratase deficiency and HLRCC syndrome. (E) Fumarate hydratase (FH) IHC: Retained expression, confirming exclusion of HLRCC. University of Michigan expert consultation confirmed no features of STUMP or leiomyosarcoma.

Post‐hysterectomy follow‐up revealed complete normalization of haematological parameters (Figure 4). By October 31, 2025—3 months after surgery—haemoglobin normalized to 14.2 g/dL, haematocrit to 42.4% and RBC to 5.21 × 10^12^/L. Serum EPO normalized to 6.7 mIU/mL (November 19, 2025). Pulmonary nodules were stable in size on follow‐up chest CT.

**FIGURE 4:**
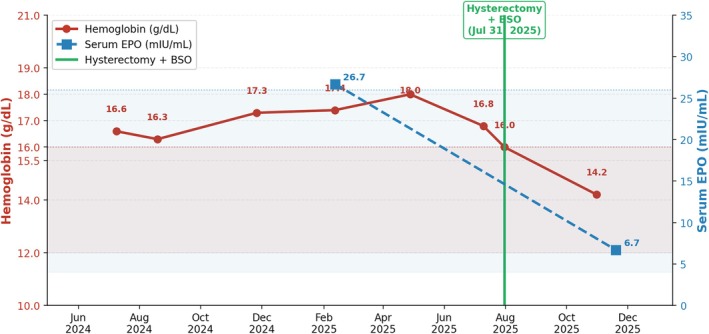
Serial haemoglobin (g/dL, red) and serum erythropoietin (EPO, mIU/mL, blue) from July 2024 through November 2025. The vertical green line marks the date of total hysterectomy with bilateral salpingo‐oophorectomy (BSO, July 31, 2025). Haemoglobin peaked at 18.0 g/dL (April 2025) with EPO elevated at 26.7 mIU/mL (February 2025). Within 3 months of surgery, both parameters normalized completely (haemoglobin: 14.2 g/dL; EPO: 6.7 mIU/mL), confirming myomatous erythrocytosis syndrome (MES)—ectopic EPO secretion by the uterine leiomyomata—as the cause of erythrocytosis.

## 3 Discussion

This case is, to our knowledge, the first reported instance of concurrent pulmonary BML and myomatous erythrocytosis syndrome (MES) presenting as a unified hormonal syndrome. The two phenomena are mechanistically distinct yet share a common substrate—uterine smooth muscle tumours—and were both resolved by a single intervention: hysterectomy with BSO.

BML is driven by sex steroid receptor signalling. ER and PR expression on lesional cells promotes proliferation and survival; oestrogen deprivation through natural or surgical menopause, GnRH agonism or aromatase inhibition consistently produces nodule stabilization or regression [4, 7]. The strong ER/PR positivity in both the pulmonary nodule (Figure 2D,E) and hysterectomy specimen is consistent with this mechanism; the post‐BSO hormonal milieu is expected to produce gradual regression of residual pulmonary disease over months to years.

MES—ectopic EPO production by uterine leiomyomata causing erythrocytosis—is confined to isolated case reports, predominantly from Japan [5, 6, 8], with the classic diagnostic triad of (1) erythrocytosis, (2) myomatous uterus and (3) normalization of haematological parameters following hysterectomy. The proposed mechanism involves hypoxia‐inducible factor (HIF) pathway dysregulation within leiomyoma tissue, leading to aberrant EPO gene expression independent of systemic hypoxia or renal pathology. In our patient, the temporal correlation between hysterectomy and complete normalization of both haemoglobin and serum EPO within 3 months (Figure 4) provides compelling evidence that the uterine leiomyomata were the source of ectopic EPO. The pre‐surgical EPO of 26.7 mIU/mL in the setting of erythrocytosis—paradoxically elevated relative to the expected suppression in primary polycythemia—was the key discriminating finding. No established ethnic or racial predisposition has been described for either BML or MES, and the predominance of MES reports from East Asia most likely reflects reporting patterns rather than true differential susceptibility.

Bilateral, well‐circumscribed pulmonary nodules in a premenopausal woman with uterine fibroids should prompt BML as a leading differential. Tissue confirmation is essential given radiographic overlap with leiomyosarcoma metastases. Comprehensive IHC including FH and 2SC to exclude HLRCC is strongly recommended. In patients with coincident MES and pulmonary BML, hysterectomy achieves dual therapeutic goals and should be considered the preferred approach when uterine preservation is not required.

The choice between surgical and non‐surgical menopause in BML warrants consideration. Non‐surgical (medical) options—GnRH agonists, aromatase inhibitors or selective oestrogen‐receptor modulators—induce a hypoestrogenic state that can stabilize or regress ER/PR‐positive pulmonary nodules while preserving the uterus and ovaries and avoiding operative risk. These therapies are, however, generally suppressive rather than curative: they require prolonged administration, carry cumulative adverse effects (vasomotor symptoms, bone mineral density loss and arthralgias), and are frequently followed by disease reactivation after discontinuation. Importantly, in the present patient, medical oestrogen suppression alone would not have eliminated the ectopic EPO source, as the erythrocytosis arose from the leiomyomata themselves rather than from hormone‐receptor signalling. Total hysterectomy with BSO therefore offered definitive, single‐procedure treatment of both processes—physically removing the EPO‐secreting uterine leiomyomata (resolving the MES) while inducing the oestrogen deprivation expected to regress the pulmonary BML. In this patient, who had completed childbearing and suffered from menorrhagia and cyclical pelvic pain attributable to her bulky myomatous uterus (563 g, > 20 nodules, 90 mm dominant fibroid), these symptoms together with the dual therapeutic imperative favoured a definitive surgical approach, which she elected following multidisciplinary counselling.

Clinicians evaluating secondary erythrocytosis (elevated EPO) with no identifiable renal, hepatic or cardiopulmonary source should include MES in the differential, particularly in premenopausal women with fibroids.

In conclusion, in this Case Report we described a novel unified hormonal syndrome in which uterine leiomyomata simultaneously drove pulmonary BML through ER/PR‐mediated signalling and caused secondary erythrocytosis (MES) through ectopic EPO secretion. Both processes were resolved following total hysterectomy with BSO, confirming the uterus as the common source. Multidisciplinary evaluation encompassing pulmonology, haematology, gynaecology, and expert pathology was essential to reach this unifying diagnosis.

## Author Contributions

**Venkatkiran Kanchustambham:** conceptualization, data curation, formal analysis, investigation, methodology, project administration, supervision, writing – original draft, writing – review and editing. **Ashley McWalter:** data curation, investigation, patient care coordination, writing – review and editing. **Oluwatobi E. Odetola:** investigation, formal analysis (haematological evaluation and polycythemia workup), writing – review and editing. **Recep Nigdelioglu:** investigation, resources (histopathological preparation, immunohistochemical staining and interpretation of all pathological specimens), visualization (pathology figures), writing – review and editing.

## Funding

The authors have nothing to report.

## Consent

Written informed consent for publication of this case report, including all accompanying clinical data, laboratory results, imaging and histopathological images, was obtained from the patient using the Respirology Case Reports patient consent form.

## Conflicts of Interest

The authors declare no conflicts of interest.

## Data Availability

The data that support the findings of this study are available on request from the corresponding author. The data are not publicly available due to privacy or ethical restrictions.
